# A Re-Evaluation of M. prototuberculosis

**DOI:** 10.1371/journal.ppat.0020098

**Published:** 2006-09-29

**Authors:** Noel H Smith

**Affiliations:** Scripps Research Institute, United States of America

It has been suggested that a group of smooth tubercle bacilli, isolated from patients with tuberculosis and associated with Djibouti, East Africa, along with the seven species and subspecies that are traditional members of the Mycobacterium tuberculosis complex, should be considered a single species. This suggestion is based on the sequence similarity of the16S rRNA and segments of six housekeeping genes [1]. The very concept of bacterial species is now subject to debate [2–5], and I follow the lead of Maynard Smith, who, in a review of the bacterial species concept, suggested that using genetic distance to define bacterial species was “arbitrary and of little merit” [6]. If defining a species by sequence diversity alone is controversial, then it is important to carefully examine the recent claim that strains of M. tuberculosis are descendants and members of a much more ancient and large bacterial species called Mycobacterium prototuberculosis [1]. Furthermore, given the importance of M. tuberculosis as a human pathogen and the implications for research [1], it is important to verify the claim that our remote hominid ancestors may have suffered from tuberculosis and that the tubercle bacilli originated in Africa [1].

Following the example of Gutierrez et al. [1], I will refer to the group of organisms associated with Djibouti in East Africa, which includes strains previously identified as *Mycobacterium canettii,* as the “smooth” group of strains, and refer to all other members of the M. tuberculosis complex, which includes *M. tuberculosis, Mycobacterium africanum, Mycobacterium pinnipedii, Mycobacterium microti, Mycobacterium caprae,* and Mycobacterium bovis as “MTBC” strains.

To demonstrate that strains of the MTBC are a subset of a larger species defined by the smooth group, Gutierrez et al. [1] have measured the average pairwise synonymous nucleotide diversity between the MTBC strains and the smooth strains and compared it with the average pairwise synonymous sequence diversity in the smooth strains alone. These authors suggest that, in all but one of the comparisons of six genes between these two groups of bacteria, the distances between MTBC alleles and the smooth alleles were within the range observed in the smooth strains alone [1]. It is the evidence from these five comparisons, and this evidence alone, that is used to suggest that M. tuberculosis is just the visible tip of a much broader species that includes all members of the M. tuberculosis complex and the smooth strains; a species the authors have named M. prototuberculosis.

If we examine the evidence that the strains of the MTBC fall within the diversity of the smooth strains, one of the six gene comparisons does not support this conclusion (*hsp*65). However, in two of the remaining five comparisons (*sodA* and *katG*), if the obvious recombinant segments present in the smooth strains were removed, then there is no synonymous divergence at all in these 17 strains. Removal of recombinant segments from *gyrB* and *gyrA* would significantly reduce levels of synonymous diversity in these genes, too. Furthermore, for the two genes (*hsp*65 and *rpoB*) without an obvious recombinant segment, *hsp*65 contradicts the authors' observation of excess diversity in the smooth group, while the evidence from the *rpoB* gene is based on a single synonymous polymorphism present in half of the smooth strains. I suggest that there is an alternative hypothesis to explain why the diversity of the MTBC strains falls within the diversity of the smooth strains—the presence of highly divergent recombinant segments in the smooth strains has generated mean divergence estimates with very similar ranges.

To test the hypothesis that the signal in the non-recombinant regions is swamped by the diversity of the recombinant segments, I simulated the evolution of two groups of sequences by mutation and then added recombinant segments to one of the groups and calculated the average pairwise divergences and ranges. In the simulation, a single ancestral sequence was used to produce two independent populations of eight sequences by replication with mutation. To simulate recombination, I changed one group of eight sequences to emulate the synonymous changes shown boxed in Figure 3 of Gutierrez et al. [1]. Then, following the protocol of Gutierrez et al. [1], I estimated both the mean pairwise divergence of the sequences containing recombinant segments (equivalent to smooth alone) and the mean pairwise divergence of the combined dataset of 16 sequences (equivalent to comparing MTBC versus smooth).

In 1,000 trials, the mean diversity of these two estimates did not significantly overlap (Figure 1A and 1B). However, in general, the pairwise comparisons of divergences had virtually identical ranges: between all 16 sequences the minimum and maximum ranges were, on average, 0.35% to 4.38% (Figure 1A), whereas the comparison of eight sequences with recombinant segments had an average range of 0.03% to 4.35% (Figure 1B). These results are similar in magnitude to those of Gutierrez et al. [1] and demonstrate that the presence of recombinant segments can generate similar ranges in comparisons of pairwise divergence of this nature.

**Figure 1:**
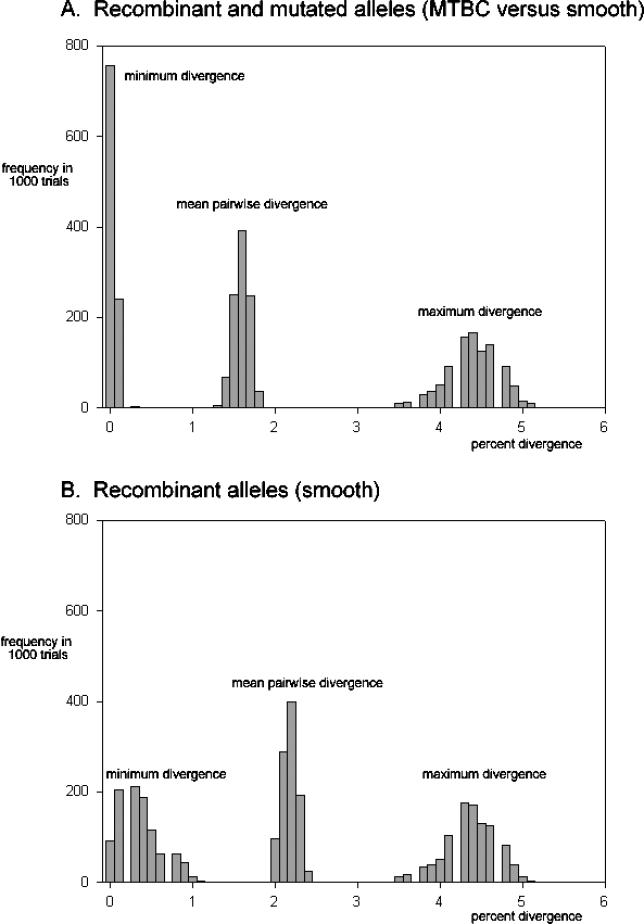
The Distribution of Mean Divergence and Maximum and Minimum Estimates of Divergence for Pairwise Comparisons of Alleles Frequency in 1,000 trials plotted against percentage divergence. (A) All alleles (representing MTBC versus smooth). (B) Recombinant alleles only (representing smooth). Percentage divergence is the equivalent of percentage divergence at synonymous sites. The simulation started with an ancestral sequence of 800 base pairs (bp), representing the synonymous sites in the 3.4 Kb of sequence presented by Gutierrez et al. [1]. This was used to generate two independent populations with eight derived sequences following three bouts of binary replication. At each replication, a randomly selected bp was chosen and, to simulate mutations, changed with probability of 0.5. This generated, on average, 15 polymorphic sites in the entire dataset of 16 derived sequences, which approximates the number of polymorphic sites in the clonal frame of the dataset presented by Gutierrez et al. [1]. To compare differences arising from recombination, only one of the two independent populations (described above) was allowed to undergo recombination. To simulate this, sets of bp were changed in proportion to the observed recombinant segments shown boxed in Figure 3 of Gutierrez et al. [1]. For example, in four randomly selected sequences, the same 5 bp were simultaneously changed to simulate the recombinant segment in *katG*. Recombinant segments did not overlap. The above simulation was run for 1,000 independent trials; starting from the same ancestral sequence and the average pairwise diversity, the maximum and the minimum pairwise diversity were recorded for each population, for each trial.

If the obvious recombinant segments are removed from the dataset of Gutierrez et al. [1], then there is very little data left to analyse, and I see no clear reason to suggest that the seven species within the MTBC are a subset of a larger species defined by the smooth group. Other, more distant strains, the donors of the recombinant segments, obviously exist [7]. The exact nature and ecology of these strains waits to be discovered, but there is no reason to believe that these unidentified donor strains cause tuberculosis in humans.

The calculation of the age of these organisms by Gutierrez et al. [1] is probably an overestimate. The primary criticism is that the vast majority of the diversity found in this group of organisms is generated by recombinant segments from unidentified strains. What the authors have attempted to calculate is the age of divergence between these unidentified strains and the M. tuberculosis complex, not the age of the most recent common ancestor of the species they have named M. prototuberculosis. This analysis could be compared with estimating the age of the human species by reference to the nuclear mitochondrial DNA [8] found in our genomes.

The divergence time estimate of Gutierrez et al. [1] suffers from a number of methodological problems, the most important of which is the application of a molecular clock based on substitution rate to polymorphism data which represents mutations rather than substitutions (substitutions are mutations that are fixed in the population) [9,10]. This will necessarily lead to an overestimation of divergence time. Other issues also need to be addressed, including the potential bias introduced by non-random sampling of strains, the relatively small number of variable sites included in the analysis [11], and the non-independence of datapoints caused by the use of all possible pairwise sequence comparisons. Furthermore, advances in molecular evolution theory and empirical evidence have shown that substitution rates are highly variable between different bacterial lineages [12,13]. Finally, we are left with the fact that the estimator used by Gutierrez et al. [1], the mean pairwise divergence of a set of 17 sequences, bears no relationship to a possible estimator of the age of these bacteria—the average number of substitutions in each sequence since the common ancestor.

If we have no accurate date for the origin of this group of organisms, then there is no compelling reason to suggest that organisms causing tuberculosis were associated with our hominid ancestors three million years ago. Furthermore, the only evidence presented in Gutierrez et al. [1] to support the African origin of *M. tuberculosis* is the excess diversity in the smooth strains isolated from East Africa compared with the sequence homogeneity of *M. tuberculosis* strains isolated worldwide. I do not find this argument persuasive. The diversity of the smooth strains can easily be explained by recombination, and I am not convinced that the worldwide distribution of smooth strains has been properly sampled. It is not that these hypotheses are impossible, just that there is no evidence to support them in the data presented by Gutierrez et al. [1].

## Summary

I do not accept the concept of M. prototuberculosis because seven well-documented species and subspecies have been described before (see references in Brosch et al. [14]), and because adding an additional 37 strains with virtually identical 16s rRNAs and very similar gene sequences should not initiate a redefinition of the whole group. I reject the hypothesis that strains of the MTBC are members of the smooth group, because the measurements of diversity are skewed by the recombinant segments identified in the smooth strains; this criticism lends further support to the rejection of the M. prototuberculosis concept. I find the calculation of the age of three million years for this group of bacteria flawed and, more important, dominated by recombinant segments in the smooth group; I see no evidence to suggest that tubercle bacilli were contemporaneous with early hominids in East Africa. The suggestion that the tubercle bacilli emerged in Africa is, in my opinion, unsupported by Gutierrez et al. [1]. However, the observation of recombination among the smooth group of strains is undeniable.

## Abbreviations

MTBC: a subgroup of the *M. tuberculosis* complex that includes *M. tuberculosis, M. africanum, M. pinnipedii, M. microti, M. caprae,* and *M. bovis*
